# Preclinical efficacy and biological effects of the oral proteasome inhibitor ixazomib in diffuse large B-cell lymphoma

**DOI:** 10.18632/oncotarget.20378

**Published:** 2017-08-21

**Authors:** Wei Liu, Juan Chen, Archito T. Tamayo, Changgeng Ruan, Li Li, Shouhao Zhou, Chan Shen, Ken H. Young, Jason Westin, Richard E. Davis, Shimin Hu, Leonard J. Medeiros, Richard J. Ford, Lan V. Pham

**Affiliations:** ^1^ Department of Hematopathology, The University of Texas MD Anderson Cancer Center, Houston, TX, United States; ^2^ Department of Lymphoma and Myeloma, The University of Texas MD Anderson Cancer Center, Houston, TX, United States; ^3^ Department of Biostatistics, The University of Texas MD Anderson Cancer Center, Houston, TX, United States; ^4^ Department of Health Services Research, The University of Texas MD Anderson Cancer Center, Houston, TX, United States; ^5^ Department of Pathology, First Affiliated Hospital of Soochow University, Suzhou, Jiangsu, China

**Keywords:** proteasome inhibitor, refractory DLBCL, ixazomib, DNA damage response, CHK2

## Abstract

Despite advances in deciphering the molecular pathogenesis of diffuse large B-cell lymphoma (DLBCL), patients with relapsed/refractory disease, particularly those with adverse genetic features (e.g., mutated p53 or double hit lymphoma (DHL)) have very poor prognoses, and effective therapies are lacking. In this study we examined the preclinical efficacy and associated biological effects of the first oral proteasome inhibitor, ixazomib, in DLBCL *in vitro* and *in vivo* models. We demonstrated that ixazomib exhibited anti-tumor activities in 28 representative DLBCL cell lines, 10 primary DLBCL samples, and a DHL xenotransplant mouse model, at clinically achievable drug concentrations. Ixazomib sensitivity in DLBCL cells is correlated with immunoproteasomal activity; stimulating lymphoma cells with interferon gamma induced immunoproteasome activity and sensitized these cells to ixazomib. In addition, we showed that ixazomib induces apoptosis and the DNA damage response pathway, through activation of the checkpoint kinase 2 (CHK2). Hence, pharmacological inhibition of CHK2 enhances the anti-tumor activity of ixazomib in DLBCL cells. Our results indicate that ixazomib is an effective proteasome inhibitor active in DLBCL, including DHL, and its combination with a CHK2 inhibitor offers a potentially more robust therapeutic regimen for treatment-resistant DLBCL.

## INTRODUCTION

Diffuse large B-cell lymphoma (DLBCL), the most common type of non-Hodgkin lymphoma, is characterized by clinical, genetic, and biological heterogeneity [1]. The current frontline chemotherapy regimen for DLBCL is R-CHOP (rituximab, cyclophosphamide, doxorubicin, vincristine, and prednisone). Using this regimen, about 60% of patients are cured with the remaining patients experiencing either refractory (∼10%) or relapse (∼30%) disease within 2–3 years. The salvage therapy options for patients with relapsed disease are poor (response rates < 20%), and survival is usually short. Notably, 2–3% of DLBCL cases with rearrangements of *MYC* and either the *BCL2* or *BCL6* gene, so-called double-hit lymphoma (DHL), are associated with the germinal center B-cell (GCB) phenotype, frequent extranodal and central nervous system involvement, higher International Prognostic Index scores, poor response to R-CHOP therapy, and overall dismal outcome [2–6]. Investigation of novel therapeutic approaches for relapsed/refractory DLBCL as well as DHL is underway, but lack of relevant human experimental models for understanding the biological basis of these cancers has hampered the identification of valid therapeutic regimens.

The ubiquitin-proteasome signaling pathway plays an important role in the proteolysis of key regulatory proteins [7, 8]. Importantly, dysregulation of this pathway is linked to the development of various diseases, including cancer, and targeting components of the pathway may offer therapeutic opportunities [8]. The development of the first-in-class proteasome inhibitor bortezomib is one of the major milestones of this approach; bortezomib is effective in the treatment of patients with new or relapsed/refractory multiple myeloma [9]. Bortezomib also inhibits cell growth and induces apoptosis in mantle cell lymphoma cells *in vitro* and has clinical efficacy in relapsed/refractory cases of this disease [10, 11]. However, the duration of response is limited, and peripheral neuropathy is a dose-limiting side effect [12, 13]. The good clinical outcome of bortezomib treatment gave impetus for the development of second-generation proteasome inhibitors, with the goals of enhancing antitumor activity and decreasing toxicity, as well as providing more flexible dosing schedules and greater patient convenience.

MLN9708 is a novel oral proteasome inhibitor that has shown promising preclinical and clinical activity in several types of cancers. Compared with bortezomib, MLN9708 is orally bioactive, has a shorter proteasome dissociation half-life and improved pharmacokinetics, and has low rates of peripheral neuropathy [14]. Upon exposure to aqueous solutions or plasma, MLN9708 immediately hydrolyzes to its biologically active boronic acid form MLN2238 (ixazomib). Ixazomib inhibits cell growth and induces apoptosis in multiple myeloma cells resistant to conventional therapies and bortezomib. Ixazomib-triggered multiple myeloma cell death has been shown to be associated with activation of caspases, activation of the p53 pathway, induction of endoplasmic reticulum stress response proteins, inhibition of NF-κB, and upregulation of miR33b [15, 16]. Several clinical trials have shown promise for ixazomib, both as a single drug and in combination with dexamethasone, in patients with relapsed/refractory multiple myeloma [17, 18]. The potential efficacy of ixazomib for treatment of refractory/relapsed DLBCL, including DHL, remains unclear.

Our purpose in the present study was to examine the antitumor activity and biological effects of ixazomib in both *in vitro* and *in vivo* models of refractory/relapsed DLBCL and DHL.

## RESULTS

### *In vitro* ixazomib sensitivity in patient-derived DLBCL cell lines

**Figure 1:**
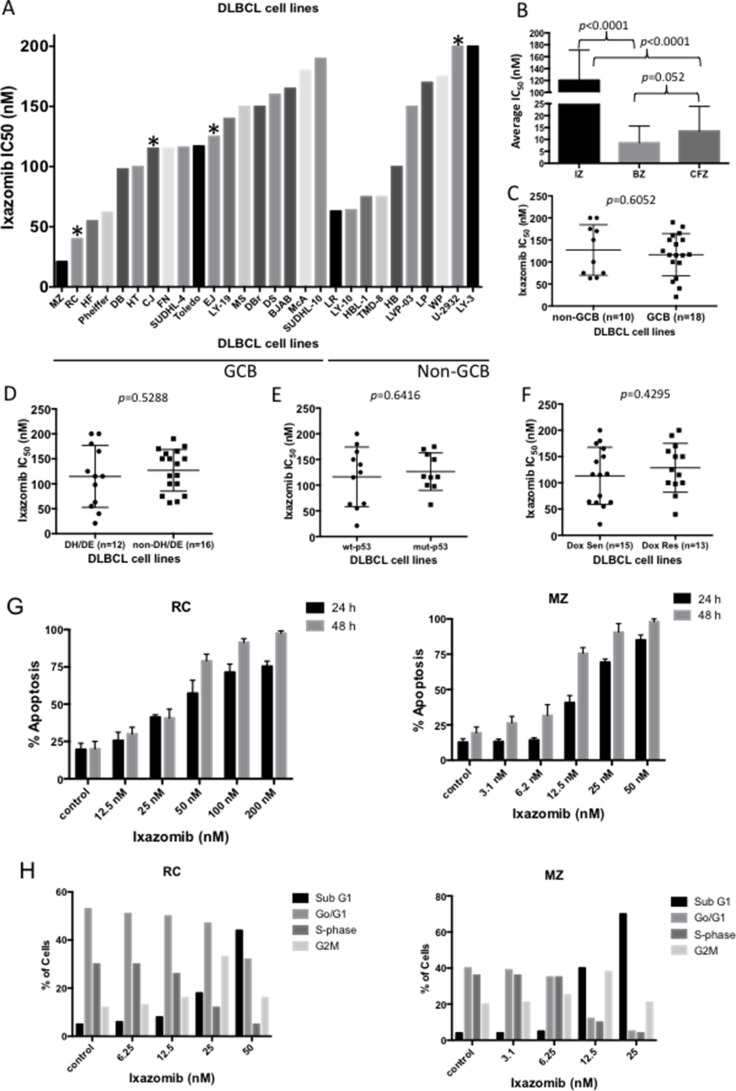
*In vitro* antitumor efficacy of the proteasome inhibitor ixazomib in DLBCL (**A**) The effect of ixazomib in a concentration-dependent manner for 72 h on viability of 28 representative DLBCL cell lines (10 non-GCB subtype and 18 GCB subtype) was assessed. The IC_50_ value for each cell line was calculated and plotted. ^*^ denotes double-hit DLBCL cell lines. (**B**) The average IC_50_ value of ixazomib (IZ) in all DLBCL cell lines was compared with those of other proteasome inhibitors such as bortezomib (BZ) and carfilzomib (CFZ). The average IC_50_ of ixazomib (120 nM) was 14-fold higher than that of bortezomib (8.6 nM) and 8.8-fold higher than that of carfilzomib (13.5 nM). (**C**) Comparison of ixazomib IC_50_ values for non-GCB (*n* = 10) and GCB (*n* = 18) DLBCL subtypes revealed no significant difference. (**D**–**F**) Comparison of ixazomib IC_50_ values for double hit/double expressor lymphoma (DH/DEL) vs. non-DH/DEL groups (D), wild-type *p53* vs. mutant *p53* groups (E), and doxorubicin-sensitive vs. doxorubicin-resistant groups (F) revealed no significant differences. (**G**) Ixazomib-sensitive DLBCL cell lines RC (left panel) and MZ (right panel) were treated with ixazomib at various concentrations for 24 or 48 h, followed by annexin V staining/FACS analysis for apoptosis identification. (**H**) RC (left panel) and MZ (right panel) cells were treated with ixazomib at various doses for 24 h and were then subjected to cell cycle analysis. Error bars indicate the standard error of the mean.

To evaluate the antitumor efficacy of ixazomib in human DLBCL, we first examined the *in vitro* effects of the drug in 28 representative DLBCL cell lines, 18 GCB and 10 non-GCB, using concentration-dependent, 72 h viability assays. Both GCB and non-GCB DLBCL cell lines showed modest responses to ixazomib, with IC_50_ values ranging from 21 to 200 nmol/L (nM) (Figure 1A; see Supplementary Figure 1 for concentration-response curves). The MZ and RC cell lines were most responsive to the drug, with IC_50_ values of 21 and 40 nM, respectively. The IC_50_ values of ixazomib in all DLBCL cell lines were then compared with those of other proteasome inhibitors such as bortezomib and carfilzomib. The average IC_50_ for ixazomib (120 nM) was 14-fold higher than that of bortezomib (average 8.6 nM) and 8.8-fold higher than that of carfilzomib (average 13.5 nM; Figure 1B). Response to ixazomib did not differ significantly between GCB and non-GCB cell lines (*p* = 0.6052; Figure 1C). Four DLBCL cell lines carried the *MYC and BCL2* translocations and met criteria for DHL [19–22]. Eight cell lines expressed both MYC and BCL-2 proteins, measured by RPPA analysis (Table 1) and therefore met the criteria for double expressor lymphoma (DEL), and 9 cell lines carried the *p53* gene mutations (Table 1). There was no significant difference in ixazomib IC_50_ values between the DH/DEL group and the non-DH/DEL groups (*p* = 0.5288; Figure 1D), nor was there a difference between the mutant *p53* and wild-type *p53* groups (*p* = 0.6416; Figure 1E), suggesting that ixazomib is also effective in poor responder DLBCL groups. Furthermore, there was no significant difference in ixazomib sensitivity between the doxorubicin-sensitive and the doxorubicin-resistant cell lines (*p* = 0.4295; Figure 1F), suggest that ixazomib is a promising therapeutic agent that has the potential to overcome treatment resistance in DLBCL. Since ixazomib effectively inhibits DLBCL cell viability, we next examined whether the drug can induce apoptosis and/or cell cycle arrest in two representative DLBCL cell lines (RC and MZ), shown to be highly sensitive to ixazomib. Cells treated with ixazomib showed both time- (24 and 48 h) and concentration-dependent increases in apoptosis (Figure 1G; see Supplementary Figure 2 for detailed histograms). Furthermore, cells treated with ixazomib accumulated in sub-G0/G1 and underwent G2M cell cycle arrest (Figure 1H).

**Table 1:** Characterization of DLBCL cell lines

	DLBCL Cell lines	Subtype	STR DNA Finger- Printing	p53 status	BCL2/MYC	IZ IC_50_ (nM)	Doxo IC_50_ (ng/mL)
1	OCI-LY10	ABC	Yes	wt	+/−	64	12.23
2	OCI-LY3	ABC	Yes	wt	+/+	200	8.45
3	SUDHL-4	GCB	Yes	mut	+/−	116	15.62
4	HF	GCB	Yes	wt	+/+	55	8.87
5	McA	GCB	Yes	wt	+/+	180	8.49
6	LR	ABC	Yes	wt	+/+	63	14.8
7	MZ	GCB	Yes	wt	+/+	21	10.4
8	FN	GCB	Yes	wt	+/+	115	12.8
9	DBr	GCB	Yes	wt	−/−	150	30.67
10	WP	ABC	Yes	mut	−/−	175	45.03
11	HB	ABC	Yes	mut	−/+	100	55.06
12	CJ	GCB (DHL)	Yes	mut	+/+	115	134.1
13	LP	ABC	Yes	mut	−/−	170	115.2
14	MS	GCB	Yes	mut	+/−	150	97.1
15	DS	GCB	Yes	mut	−/−	160	122.2
16	EJ	GCB (DHL)	Yes	wt	+/+	125	65.2
17	Toledo	GCB	Yes	wt	+/−	117	12
18	Pfeiffer	GCB	Yes	mut	−/−	62	50
19	OCI-LY19	GCB	Yes	wt	+/+	140	20
20	BJAB	GCB	Yes	wt	−/+	165	22
21	U2932	ABC (DHL)	Yes	ND	+/+	200	75
22	RC	GCB (DHL)	yes	ND	+/+	40	95
23	LVP-03	PM-BCL	yes	ND	−/−	150	98
24	HBL-1	ABC	Yes	ND	+/−	75	65
25	TMD-8	ABC	Yes	ND	−/+	75	20
26	DB	GCB	Yes	mut	+/+	98	60
27	HT	GCB	Yes	ND	−/−	100	85
28	SUDHL-10	GCB	Yes	ND	+/−	190	120

ABC and GCB-DLBCL subtypes of the cell lines were previously characterized. All cell lines were authenticated using short tandem repeat DNA fingerprinting at the Characterized Cell Line Core Facility at The University of Texas MD Anderson Cancer Center. BCL-2 and MYC protein expressions were determined by reverse-phase protein array and validated by Western blotting. All cells were collected at log phase before protein purification. The cut-offs were determined by log2 protein expression level, values below zero were considered negative and above zero were considered positive. Ixazomib (IZ) and Doxorubicin (Dox) IC_50_ values for each cell line were determined using a dose-dependent 72 h viability assay. For Doxorubicin, cell lines with IC_50_ values below 50 ng/ml were considered “Dox Sensitive” and cell lines with IC_50_ values of 50 ng/ml and above were considered “Dox Resistant”. ABC, activated-like B cell; GCB, germinal center B cell; DH, double hit; PM-BCL, plasmablastic B cell lymphoma; STR, short tandem repeat; wt, wild type; mut, mutant; ND, no data; IZ, ixazomib; Doxo, doxorubicin.

### *In vitro* effects of ixazomib in primary DLBCL cells and *in vivo* efficacy of ixazomib in a DHL xeno-transplant NOD SCID gamma (NSG) mouse model

**Figure 2:**
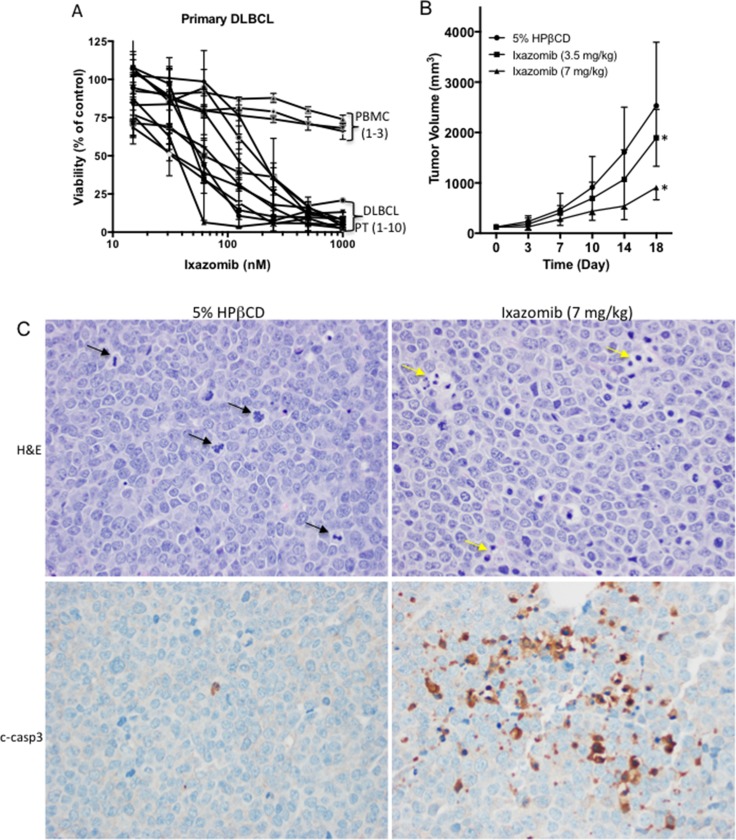
*In vitro* effects of ixazomib in primary DLBCL cells and *in vivo* efficacy of ixazomib in a DHL xeno-transplant mouse model (**A**) The effect of ixazomib at various doses for 72 h was assessed by viability assay in primary DLBCL cells from 10 representative patients and peripheral blood mononuclear cells (PBMC) isolated from 3 healthy donors. (**B**) The effects of ixazomib on tumor growth in NOD-SCID mice with established RC tumors are shown. The graph describes the change in tumor volume as a function of the time after initiation of treatment. Each point on the curves represents the mean of 10 tumors. Error bars depict the SEMs. Ixazomib was administered orally twice a week. A 5% solution of HPβCD was used as the vehicle control. ^*^p-value < 0.001. (**C**) Tumor tissues from control and ixazomib-treated mice were fixed, sectioned, and stained for H&E (top panels) or cleaved caspase-3 (bottom panels). 40×; black arrows pointing to mitotic cells; yellow arrows pointing to apoptotic bodies.

To assess the effects of ixazomib on the viability of fresh primary human DLBCL cells, lymphoma cells were isolated from a panel of 10 DLBCL patients who had been exposed to various standard therapies (Table 2). The primary cells were treated with ixazomib in a concentration-dependent manner and the observed median IC_50_ values ranged from 36–200 nM (Figure 2A and Table 2), similar to the range found in DLBCL cell lines *in vitro*. Notably, the similar range of drug concentrations of ixazomib did not affect proliferation of resting PBMCs from three healthy volunteers (Figure 2A). These results indicate that ixazomib selectively inhibits proliferation of primary DLBCL cells without affecting the growth of normal lymphocytes. To determine whether the *in vitro* effects of ixazomib on DLBCL cells translate to an *in vivo* setting, we evaluated the ability of ixazomib to suppress tumor growth *in vivo* in a DHL xeno-transplant model established in NSG mice. Treatment of tumor-bearing mice with ixazomib 3.5 mg/kg or 7 mg/kg body weight orally twice a week for 2 weeks resulted in significant reduction of tumor burden (Figure 2B). The magnitude and durability of the response increased with the dose; treatment with 7 mg/kg conferred a TGI_max_ of 64% (*p* < 0.001). Due to the aggressiveness of the tumor in the control group, the experiment was terminated after the fifth treatment and all mice in the control and ixazomib 3.5 mg/kg treatment groups were sacrificed. Mice in the ixazomib 7 mg/kg treatment group were also sacrificed within the next 10 days due to excessive tumor burden. A representative tumor tissue sectioned from an ixazomib-treated mouse show increased caspase-3 activation in comparison to a tumor tissue sectioned from a control untreated mouse, indicative of cells undergoing apoptosis *in vivo* after ixazomib treatment (Figure 2C). In addition, more cells were found in the mitotic phase of the cell cycle in control vs. ixazomib-treated mice (Figure 2C and Supplementary Figure 3), further validating our *in vitro* findings showing ixazomib induces cell cycle arrest.

**Table 2:** Patients with refractory DLBCL

DLBCLPatient	Sex	Age	COO	Treatment	Response	Status	Sample source	OS (months)	IZ IC_50_nM
PT-1	M	46.9	Non-GCB	RICE, splenectomy	progression	Dead	Fluid	6.5	59.2
PT-2	M	82.5	Non-GCB	R-CHOP	relapse and progression	Dead	PB	13.5	57.9
PT-3	F	48.2	Non-GCB	R-ESHAP, hyper- CVAD, auto SCT	relapse and progression	Dead	Fluid	14.8	35.9
PT-4	F	75.6	Non-GCB	R-CHOP, R-ESHAP, Rituxan+MTX +cytarabine, COPP, radiaRon, Revlimid	partial remission and progression	Dead	Apheresis	40.7	122
PT-5	M	55.5	GCB	CHOP, hyper-CVAD, RICE, RDAP	relapse and progression	Dead	PB	14.4	200
PT-6	M	47.4	Non-GCB	CHOP, DA-EPOCH, ICE, auto SCT, hyper- CVAD	partial remission and progression	Dead	Apheresis	29.2	201
PT-7	M	38.7	GCB	R-CHOP, RICE	progression	Dead	Fluid	8.2	132
PT-8	F	69.0	GCB	R-hyper-CVAD, R- EPOCH, RICE, ritumimab_revlimid	progression	Dead	Fluid	49.3	60.8
PT-9	F	68.2	GCB	R-CHOP, R-GEMOX, benamusRne +rituximab	progression	Dead	Apheresis	38.1	44.5
PT-10	M	63.5	GCB	R-CHOP, RICE, auto SCT, R-GEMOX	relapse and progression	Dead	PB	10.2	75

Dx, diagnosis; COO, cell of origin; OS, overall survival; IZ, ixazomib; NA, not available; GCB, germinal center B cell; RICE: rituximab, ifosfamide, carboplatin, etopiside; VTEP, R-CHOP: rituximab-cyclophophamide, hydroxydaunorubicin, oncovin, prednisone; R-ESHAP: rituximab-etoposide, solu-medrol-methylprednisolone, high-dose Ara-C-cytarabine, platinol; CVAD: cyclophosphamide, vincristine, doxorubicin, dexamethasone; DA-EPOCH: dose adjusted-etoposide, prednisolone, oncovin, cyclophosphamide, hydroxydaunorubicin; R-GEMOX: rituximab-gemcitabine, oxaliplatin; SCT, stem cell transplant; PB, peripheral blood.

### Correlation between proteasomal activities and ixazomib sensitivity in DLBCL

**Figure 3:**
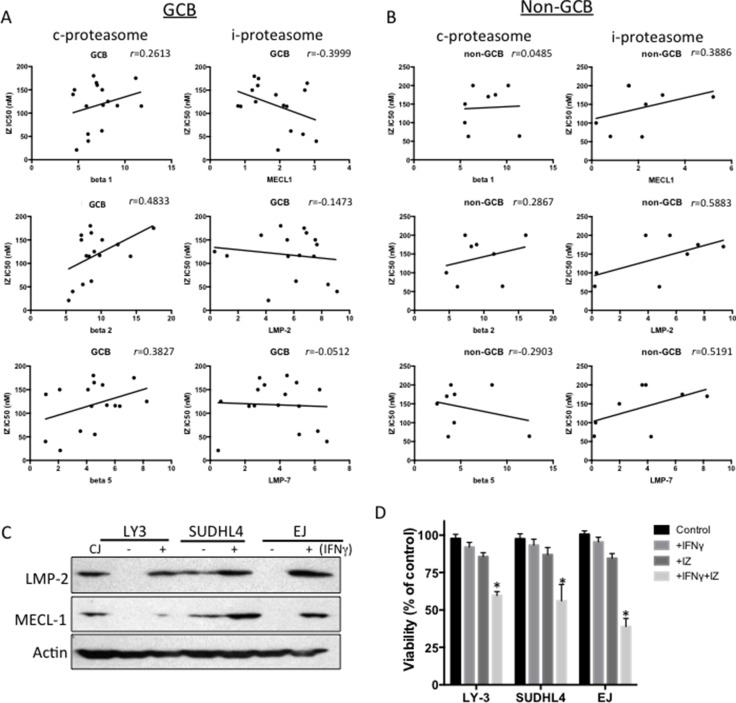
Correlation between proteasomal activities and ixazomib sensitivity in DLBCL (**A**, **B**) Proteasomal activities were assessed by the ProCISE assay and plotted against the corresponding ixazomib IC_50_ for GCB (A) and non-GCB (B) DLBCL cell lines. Correlation coefficients were determined by the Pearson rank correlation and one-tailed *t*-test. (**C**) Three DLBCL cell lines (OCI-LY3, SUDHL-4, and EJ) with low/negative activity of i-proteasome subunits LMP2 and MECL-1 were stimulated with IFN-γ for 48 h and the cell extracts were subjected to western blotting for LMP-1 and MECL-1. Actin was used as a loading control. The CJ cell line was used as a positive control. (**D**) The same three DLBCL cell lines (OCI-LY3, SUDHL-4, and EJ) with low/negative activity of i-proteasome subunits LMP2 and MECL-1 were stimulated with IFN-γ (100 units/mL) for 48 h and then treated with ixazomib (IZ; IC_25_ concentrations) for another 48 h and cell viability was assessed.^*^p-value < 0.05.

To determine whether proteasome activity can predict ixazomib sensitivity, we utilized the ProCISE assay to quantify proteasome subunit occupancy in 24 representative DLBCL cell lines (16 GCB and 8 non-GCB). In general, constitutive (c)-proteasome and immuno (i)-proteasome activity were detected in most of the DLBCL cell lines, although i-proteasome activity was low in some cell lines (Supplementary Figures 4 and 5). The sensitivity trend of ixazomib positively correlated with activity of the beta2 subunit of the c-proteasome in all DLBCL cell lines, where cell lines with lower beta2 subunit is more sensitive to ixazomib treatments (Table 3). However, when the cell lines were stratified into GCB and non-GCB subsets, in ixazomib-treated cells the GCB subset showed a trend toward a positive correlation for the c-proteasome subunits and a negative correlation for the i-proteasome subunits, particularly MECL-1 (Figure 3A). In non-GCB cells, there was a trend for positive correlation with i-proteasome subunits (Figure 3B). These results suggest that GCB cells with higher i-proteasome subunit activity are more sensitive to ixazomib, whereas non-GCB cells with higher i-proteasome subunit activity are more resistant to the drug. Since i-proteasome subunits can be induced by interferon-gamma (IFN-γ), we selected three DLBCL cell lines that were deficient or had low expression of both the LMP-2 and MECL-1 subunits and stimulated them with IFN-γ. After 48 h of IFN-γ exposure, the LMP-2 and MECL-1 subunits were induced (Figure 3C) and the cells were sensitized to ixazomib (Figure 3D).

**Table 3:** Proteasomal activity vs. ixazomib sensitivity in DLBCL

All	Correlation Coefficient (r)	*p* value
Beta 1	0.2009	0.3467
Beta 2	0.**402**	**0.0515**
Beta 5	0.1037	0.6298
MECL1	0.04508	0.8343
LMP2	0.1211	0.5728
LMP-7	0.1874	0.3806
**GCB**	**Correlation Coefficient (r)**	***p* value**
Beta 1	0.2613	0.3284
Beta 2	**0.4833**	**0.0579**
Beta 5	0.3827	0.1435
MECL1	**−0.3999**	**0.1248**
LMP2	−0.1473	0.5861
LMP-7	−0.05122	0.8506
**Non-GCB**	**Correlation Coefficient (r)**	***p* value**
Beta 1	0.04858	0.9091
Beta 2	0.2867	0.4912
Beta 5	−0.2903	0.4855
MECL1	0.3886	0.3413
LMP2	0.5883	0.1251
LMP-7	0.5191	0.1874

### Proteomic analysis of ixazomib-treated DLBCL cells

**Figure 4:**
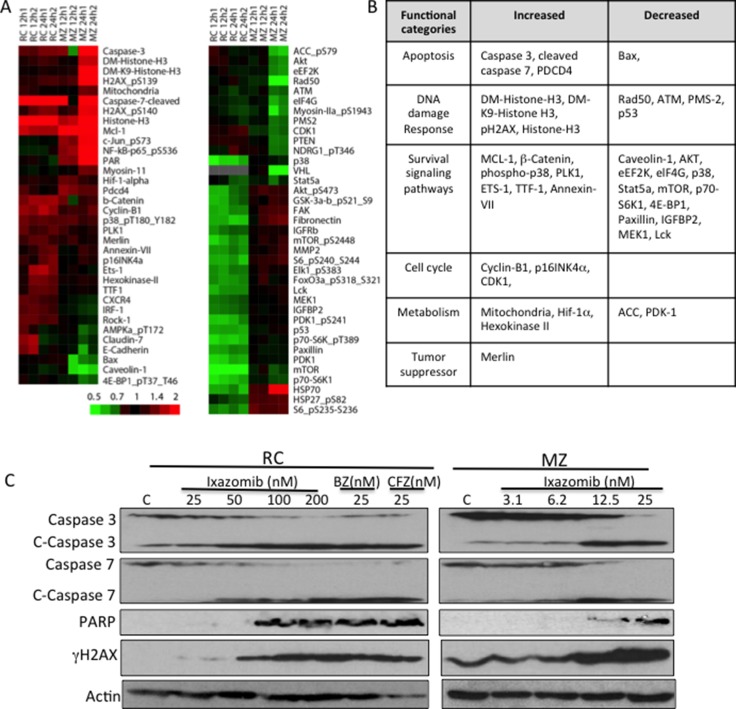
Proteomic analysis in control and ixazomib-treated DLBCL cells Ixazomib-sensitive RC and MZ DLBCL cells were treated with ixazomib (IZ; at IC_75_ concentrations, 50 nM and 30 nM, respectively) for 12 or 24 h. Protein extracts from treated cells were subjected to reverse-phase protein array (RPPA) analysis. (**A**) “Subtracted” heat maps of RC and MZ lines for antibodies whose RPPA log2 values changed with IZ treatment by at least 0.4 from control in at least 4 of the 8 time points. The color bar shows the fold-change from control. (**B**) Table showing the overlapped increased and decreased proteins in both RC and MZ cells after ixazomib treatment. (**C**) RC and MZ cells were treated with various concentrations of ixazomib. RC cells treated with bortezomib (BZ) and carfilzomib (CFZ) were used as controls. Protein extracts from treated cells were subjected to western blotting for caspase 3, caspase 7, cleaved PARP, γH2AX, and actin (loading control).

To further gain mechanistic insights into the response of DLBCL to ixazomib, we utilized a proteomics approach via RPPA in two representative DLBCL cell lines that were shown to be highly sensitive to ixazomib. RC and MZ cells were treated with ixazomib at IC_75_ concentrations at different time intervals (12 and 24 h), and then proteins were extracted and purified from control and treated cells and cellular protein level changes were measured across a set of 285 antibodies. As shown in the “subtracted” heat maps (Figure 4A), a total of 71 proteins were altered by a log2 fold change of > 0.4 in at lease one comparison with IZ-treated RC and MZ cells. Of the 71 proteins, 41 (58%) were found overlapping, either up (20)- or down (21)- regulated in both RC and MZ cells treated with IZ (Figure 4B); these proteins are involved in various cellular processes, including apoptosis (caspases 3,7 and bax), DNA damage (γH2AX, Histone 3), growth/survival signaling (AKT, MCL-1, Stat5a), cell cycle regulation (p16INK4a), metabolism (Mitochondria/Hexokinase II), and tumor repressor (Merlin) (Figure 4B). There were some variations between the two cell lines. Nine proteins were found induced in RC cells after IZ treatment but were found down-regulated in MZ cells after IZ treatment. On the other hand, 20 proteins were found induced in MZ cells but were found down-regulated in IZ-treated RC cells. Using Western blot analysis, we confirmed that ixazomib induces apoptosis through activation of caspases 3 and 7 and cleavage of PARP (Figure 4C). Induction of γH2AX by ixazomib in both RC and MZ cells (Figure 4C) suggests that the drug can induce DNA damage in DLBCL cells.

### Synergism of ixazomib with inhibition of the DNA damage repair pathway through CHK2 to induce robust DNA damage and apoptosis

**Figure 5:**
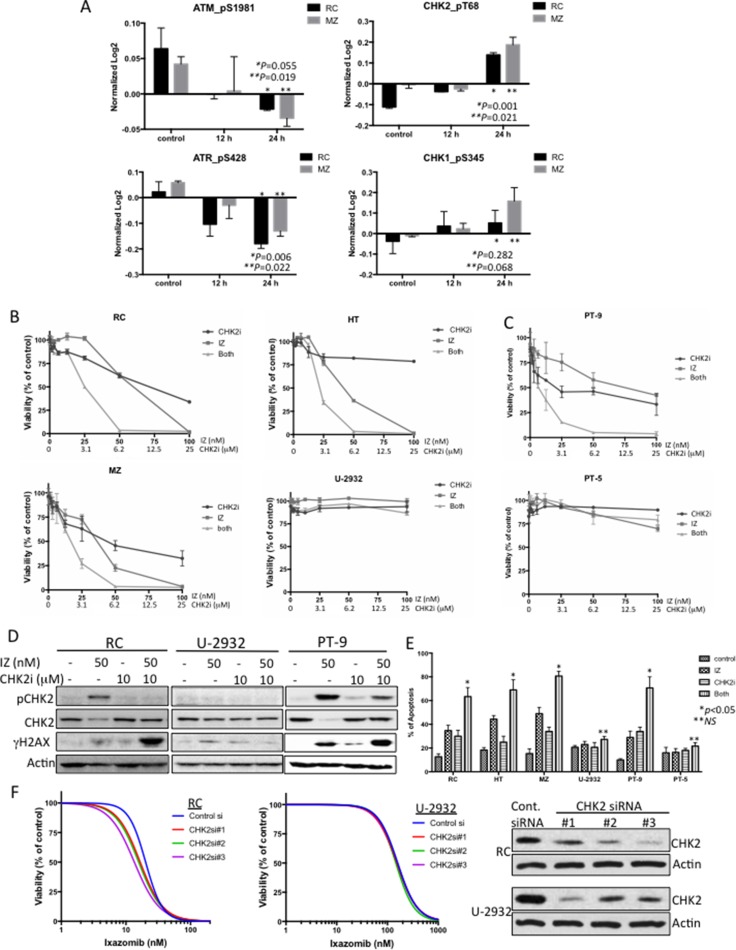
Synergism of ixazomib with inhibition of the DNA damage repair pathway through CHK2 to induce robust DNA damage and apoptosis (**A**) Protein level changes of pATM, pATR, pCHK1 and pCHK2 in control and IZ-treated RC (black bars) and MZ (gray bars) cells for 12 and 24 h. Data for these proteins did not showed up in the heat maps because they were below the 0.4-cutoff threshold. (**B**) Representative ixazomib-sensitive (RC, MZ, and HT) and ixazomib-resistant (U-2932) DLBCL cell lines were co-treated with ixazomib (IZ) and a CHK2 inhibitor (CHK2i) at concentration-dependent manner for 72 h and cell viability was assessed. Data from two independent experiments performed in triplicate are shown. (**C**) Primary DLBCL cells from two representative patients (PT-9 [ixazomib-sensitive] and PT-5 [ixazomib-resistant]) were co-treated with ixazomib and CHK2i at various doses for 48 h, and cell viability was assessed. Data from two independent experiments performed in triplicate are shown. (**D**) RC, U-2932, and primary DLBCL (PT-9) cells were treated with ixazomib (50 nM) alone, CHK2i (10 µM) alone, or combination of both drugs for 24 h. Protein extracts from the cells were subjected to western blot analysis to detect pCHK2, CHK2, γH2AX (a DNA damage marker), and actin (protein loading control). (**E**) Representative ixazomib-sensitive (RC, MZ, HT, and PT-9) and ixazomib-resistant (U-2932 and PT-5) DLBCL cells were treated with ixazomib (50 nM) alone, CHK2i (10 µM) alone, or combination of both drugs for 24 h. Apoptotic cells were detected by annexin V/propidium iodide staining. Data shown represent means ± SD from three independent experiments. (**F**) RC and U-2932 cells were transiently transfected with control or CHK2 siRNA. At 48 h post-transfection, protein purified from transfected cells was subjected to western blotting for CHK2 and actin (loading control). Small portion of the transfected cells were treated with increasing concentrations of ixazomib for an additional 72 h and viability was assessed. Data are expressed as means ± standard deviation of at least 2 independent experiments.

Since ixazomib was shown to induce a robust DNA damage response in DLBCL cells, we evaluated whether the DNA damage repair (DDR) pathways were induced in ixazomib-treated DLBCL cells. As shown in Figure 5A, pATM and pATR proteins were inhibited and pCHK2 and pCHK1 were significantly induced in cells treated with ixazomib. Since CHK2 shows a more significant increased in both cell lines tested, we examine whether blocking ixazomib-induced pCHK2 would lead to a more robust antitumor response in DLBCL cells. As expected, ixazomib in combination with a CHK2 inhibitor (CHK2i) showed synergistic activity in DLBCL cell lines that were more sensitive to ixazomib (RC, MZ, and HT), but not in cells that were more resistant to ixazomib (U-2932) (Figure 5B; see Supplementary Figure 4 for detailed synergy calculations). Similar results were obtained in primary DLBCL cells from two patients, one sensitive (PT-9) and one resistant (PT-5) to ixazomib (Figure 5C). CHK2i inhibited ixazomib-induced CHK2 phosphorylation in ixazomib-sensitive RC and PT-9 cells, but not in ixazomib-resistant U-2932 cells (Figure 5D). Similarly, combined treatment with ixazomib and CHK2i caused a striking increase in γH2AX (DNA damage) in DLBCL cell line (RC) and primary (PT-9) cells but not in U-2932 cells (Figure 5D), and increase in cells undergoing apoptosis as compared with either agent alone (Figure 5E). Furthermore, these results were independently confirmed using siRNA-mediated knock-down of CHK2 in RC and U-2932 cells, which shows that CHK2 silencing sensitized ixazomib-sensitive cells (RC) but not ixazomib-resistant cells (U-2932) (Figure 5F). These findings confirm that co-administration of a CHK2i markedly potentiates DNA damage in DLBCL cells exposed to ixazomib and that this event precedes induction of extensive apoptosis.

## DISCUSSION

Although earlier studies have shown that the novel proteasome inhibitor ixazomib has *in vitro* and vitro antitumor activity in DLBCL, its efficacy in high-risk DLBCL, including DHL, has yet to be examined [14, 23]. In the present study, we further show the following: (i) ixazomib can effectively kill refractory/relapsed GCB and non-GCB DLBCL cells, as well as DHL cells in *in vitro* and *in vivo* models; (ii) ixazomib has low toxicity in normal PBMCs; (iii) ixazomib sensitivity correlates with immunoproteasome activity, particularly in the GCB subtype of DLBCL; (iv) ixazomib induces DNA damage and apoptosis in DLBCL cells; and (v) a CHK2 inhibitor suppresses ixazomib-induced CHK2 phosphorylation, resulting in a synergistic inhibition of cell growth and DNA damage.

Bortezomib was the first proteasome inhibitor that was approved by the United States Food and Drug Administration for the treatment of patients with multiple myeloma and relapsed mantle cell lymphoma [24, 25]. Dunleavy and colleagues showed that single agent bortezomib has minimal activity in DLBCL, but enhances the activity of standard chemotherapy regimens in patients with ABC (non-GCB) subtype of DLBCL [26]. Therefore, patients with relapsed/refractory non-GCB DLBCL may benefit from treatment with a combination of proteasome inhibitors and other chemotherapy regimen [26, 27]. Here, we show that ixazomib is active in many DLBCL cell lines of both GCB and non-GCB subtype. In addition, we show that ixazomib is active against *MYC*/*BCL2* DHL, MYC/BCL2 DEL cell lines, mutant *p53* DLBCL cell lines, and doxorubicin-resistant DLBCL cell lines. These results show that ixazomib is also active in high-risk DLBCL groups, extending beyond subtypes tested in previous studies [14, 28]. Furthermore, ixazomib inhibited viability of fresh primary DLBCL cells derived from 10 patients with treatment-refractory disease, but had little effect on normal PBMCs, indicating that the drug is effective but safe for DLBCL patients.

In this study we have shown that the IC_50_ of ixazomib is significantly higher than that of other proteasome inhibitors such as bortezomib and carfilzomib. This difference may be due to ixazomib having a shorter proteasome dissociation half-life than bortezomib, [29] so that a higher concentration of ixazomib is needed to attain an equivalent effect. Clinical pharmacokinetic studies estimated a plasma C_max_ of 150 nM from once-weekly orally administered ixazomib, [30] higher than the average IC_50_ of 120 nM in our study. Our finding that ixazomib was very effective in a DHL xeno-transplant mouse model, significantly decreasing tumor burden, suggest that this drug may be promising for the treatment of patients with DHL, a group of patients with a treatment resistant tumor and very poor prognosis, as well as patients with refractory DLBCL overall. This observation is also in line with reports that ixazomib had significantly less severe side effects than bortezomib and displayed a manageable safety profile in clinical trials of heavily pretreated patients who had refractory myeloma [17, 18]. The mechanism(s) for ixazomib effectiveness in high risk DLBCL subtypes such as DHL is still unknown, but could possibly be due to higher immunoproteasome activity in these cells, as our study shows that ixzomib treatments were more responsive to cells with higher immunoproteasome subunit MECL1 activity, particularly in the GCB-DLBCL subtype. Further studies are required to validate these findings.

In an RPPA analysis performed on two representative cell lines, ixazomib consistently induced DNA damage through upregulation of the DNA damage marker γH2AX. To mitigate DNA damage, ixazomib-treated cells also activated DNA damage response (DDR) pathways by activating CHK1/CHK2 pathway. Two major signaling pathways drive DDR, ATM/CHK2 for DNA double-strand breaks and ATR/CHK1 for single-stranded DNA breaks. Inactivation of the DDR pathways has been shown to efficiently sensitize malignant cells to radiotherapy and chemotherapy [31, 32]. In addition, therapeutic targeting of CHK2 in the DDR pathway has shown to be feasible in DLBCL [33, 34]. Interestingly, our findings show that while pCHK2 was induced in ixazomib-treated cells, the level of phosorylated ATM was inhibited, suggesting that the activation of CHK2, and probably CHK1, is independent of ATM and ATR activation, respectively. This could be due to stimulation by different kinases, such as DNA-PK, which is known to activate CHK2 independent of ATM, [35, 36] or protein accumulation due to proteasomal inhibition, as the turnover of many of these DNA damage-associated proteins is controlled by the 26S proteasome [37]. Our results also show that a CHK2 inhibitor blocked ixazomib-induced CHK2, synergistically inhibiting cell growth, inducing apoptosis, and increasing DNA damage, confirming that abrogation of the CHK2 DDR in ixazomib-treated cells will lead subsequently to more robust DNA damage. These findings suggest that targeting CHK2, and probably CHK1, in combination with proteasome inhibition could be an innovative strategy to overcome chemoresistance in patients with DLBCL [38].

Taken together, our results demonstrate the preclinical efficacy and biological effects of ixazomib in *in vitro* and *in vivo* DLBCL models. To the best of our knowledge, this is the first report showing that ixazomib is an effective proteasome inhibitor active in high-risk DLBC, including DHL, and this drug therefore has the potential to become an effective agent in the treatment of patients with DLBCL refractory to standard therapy. Our data provide pre-clinical evidence to support the clinical development of ixazomib, probably in combination with novel check-point kinase inhibitors, for the treatment of patients with DLBCL, particularly refractory DLBCL.

## MATERIALS AND METHODS

### Cells and reagents

DLBCL cell lines MS, DS, DBr, JM (McA), FN, EJ, HF, HB, MZ, LR, CJ, LP, WP, LVP-03, and RC were established in our laboratory and were characterized and described previously [19, 39]. The Pfeifer and BJAB DLBCL cell lines were purchased from ATCC (Manassas, VA). The DLBCL cell lines U-2932, OCI-LY19, Toledo, SUDHL-4, SUDHL-10, HBL-1, TMD-8, DB, HT, OCI-LY10, and OCI-LY3 were obtained from Drs. Michael Rosenblum and Eric Davis (UT MD Anderson Cancer Center). All cell lines were routinely tested for mycoplasma using a Myco Tect kit (Invitrogen, Carlsbad, CA) and were validated by short tandem repeat DNA fingerprinting at the Characterized Cell Line Core Facility at The University of Texas MD Anderson Cancer Center. Stocks of authenticated cell lines were stored in liquid nitrogen for future use, and all cell lines used in the studies described here were from these authenticated stocks and were not passage more than six months from the time of thawing.

Primary DLBCL cells were isolated from samples obtained from patients through a protocol approved by the Institutional Review Board at MD Anderson Cancer Center. Normal peripheral blood mononuclear cells (PBMCs) were isolated from blood samples from healthy volunteers using the human B-cell enrichment cocktail from StemCell Technologies (Vancouver, BC, Canada) and used as lineage/cellular controls. Informed consent was obtained from all donors. The cells were cultured in RPMI medium (Gibco, Rockville, MD) containing 15% fetal calf serum (Gibco) and 1% penicillin/streptomycin (Hyclone, Logan, UT).

MLN9708 (ixazomib citrate, ixazomib) and bortezomib drug stocks were provided by Millennium Pharmaceuticals, Inc., a subsidiary of Takeda Pharmaceutical Company Limited (Cambridge, MA). Carfilzomib was provided by Amgen (Thousand Oaks, CA). CHK2 inhibitor was purchased from Sigma Aldrich (St. Louis, MO).

### Antibodies and siRNA

Monoclonal and polyclonal antibodies specific for the following molecules were used: γ-H2AX, caspase 3, c-caspase 3, caspase 7, c-caspase 7, CHK2, and pCHK2 (Cell Signaling Technology, Danvers, MA); LMP-2, MECL-1, and PARP (Santa Cruz Biotechnology, Santa Cruz, CA). Pre-designed and validated CHK2 siRNA (S22119, S22120, S22121) and control siRNA were purchased from ThermoFisher Scientific (Waltham, MA).

### Viability assays

Cells from representative DLBCL cell lines were plated at 5,000 cells per well in 384-well plates. The cells were incubated for 72 hours (h) in 20 µL medium containing 15% fetal bovine serum and the indicated drug at various concentrations or dimethylsulfoxide (DMSO) as control. Viability was assessed with the Celltiter-Glo Luminescent Cell Viability Assay according to the manufacturer’s instructions (Promega, Madison, WI). To determine the half maximal inhibitory concentration (IC_50_), six to eight concentrations in a sequence of two-fold increases were chosen for each cell line so that the IC_50_ point was approximately in the middle of the concentration range. The experiments were performed at least two times independently; in every experiment, each concentration was tested in triplicate samples.

Western blotting, apoptosis, cell cycle analysis, and transient transfection methods were performed as previously described [40, 41].

### *In vivo* ixazomib treatment in a xeno-transplant DHL mouse model

All animal experiments were reviewed and approved by the MD Anderson Institutional Animal Care and Use Committee (IACUC; protocol #: 00001480-RN00). Six-week-old female severe combined immunodeficient (SCID) NOD.Cg-Prkdcscid Il2rgtm1Wjl/SzJ mice were purchased from Jackson Laboratories (Bar Harbor, ME) and housed under specific pathogen-free conditions at the SCID Mouse Barrier Facility at MD Anderson. A recently established DHL cell line, RC, was used for the *in vivo* study [21]. RC cells (1 × 10^7^ in 100 µL phosphate-buffered saline solution [PBS]) were injected intraperitoneally into the right abdomen of each mouse. After 3 weeks, when the tumors were palpable (approximately 125 mm^3^), mice were randomly divided into three groups (*n* = 10/group), and the drug treatment was started that day (day 0). Mice were treated with ixazomib (3.5 mg/kg or 7 mg/kg body weight) or with vehicle control (5% 2-hydroxypropyl-β-cyclodextrin (HPβCD)), as previously described, [23] in a 100-µL volume via oral gavage twice a week (Monday and Thursday) for 2 weeks. Ixazomib was formulated for oral dosing in 5% HPβCD. Electronic calipers were used to measure the length and width of each tumor twice a week. Tumor volume was calculated by applying the following equation: tumor volume = length × width^2^/2. Maximum tumor growth inhibition (TGI_max_) was calculated as the greatest treatment response using the following equation: TGI_max_ = (1 − mean tumor volume of the treated group/mean tumor volume of the vehicle control group) × 100. The animals were sacrificed when tumor reached protocol limit, ∼2,500 mm^3^. Tumors were removed and the animal carcasses disposed of according to IACUC guidelines.

### ProCISE assay

The proteasome constitutive/immunoproteasome subunit enzyme-liked immunosorbent (ProCISE) assay was carried out in 24 representative DLBCL cell lines (16 GCB and 8 non-GCB), using a previously published methodology [42].

### Reverse-phase protein array

Reverse-phase protein arrays (RPPA) were analyzed and antibodies validated by the RPPA Core Facility at MD Anderson [43]. Total protein lysates were prepared by resuspending cells in lysis buffer (1% Triton X-100, 50 mM HEPES [pH 7.4], 150 mM NaCl, 1.5 mM MgCl2, 1 mM EGTA, 100 mM NaF, 10 mM NaPPi, 10% glycerol, 1 mM PMSF, 1 mM Na_3_VO_4_, and 10 μg/mL aprotinin). Protein lysates were adjusted to a 1 μg/μL concentration, and a serial dilution of five concentrations was printed on nitrocellulose-coated slides (Grace Bio-Labs, Bend, OR) with 10% of the samples replicated for quality control (2470 Arrayer; Aushon Biosystems, Billerica, MA). Immunostaining was performed using a DakoCytomation-catalyzed system with the diaminobenzidine colorimetric reaction (Dako, Carpenteria, CA). Slides were scanned on a flatbed scanner to produce 16-bit tiff images. Spot intensities were analyzed and quantified using an Array-Pro Analyzer (Meyer Instruments, Houston, TX) to generate spot signal intensities. Overall, 285 unique antibodies and 4 secondary antibody negative controls were analyzed. The Gene Cluster 3.0 (http://cluster2.software.informer.com/3.0/) software was used for data analysis, and the heat map was created by Java TreeView (http://rana.lbl.gov/EisenSoftware.htm) software.

### Statistical analysis

The IC_50_ values were calculated for each cell line using CalcuSyn software (Biosoft, Cambridge, MA). The Pearson rank correlation coefficient was used to evaluate the correlation between proteasome inhibitor IC_50_ values and proteasome enzymatic activity. Asymptotic *t* approximation was used for computation of its *p*-value. Relative protein levels for each sample in the RPPA analysis were determined by interpolation of each dilution curve from the “standard curve” using R package *SuperCurve*. All data points were normalized for protein loading and transformed to linear values for bar graphing. Normalized linear values were transformed to log2 values and then median-centered for hierarchical cluster analysis and for heatmap generation. The heatmap was generated in Cluster 3.0 (http://cluster2.software.informer.com/3.0/) as a hierarchical cluster using Pearson correlation and a center metric, visualized in Treeview (http://rana.lbl.gov/EisenSoftware.htm), and presented in a high-resolution .bmp format. Significance in the experiments for TGI_max_ was determined by the Student *t*-test and Wilcoxon rank sum test, respectively. Significance for complete response was determined by a two-tailed Fisher exact test. *P*-values of < 0.05 is considered statistically significant.

## SUPPLEMENTARY MATERIALS FIGURES



## Abbreviations

DLBCL: diffuse large B cell lymphoma
DHL: double-hit lymphoma
RPPA: revers-phase protein array
CHK2: check-point kinase 2
R-CHOP: rituximab, cyclophosphamide, doxorubicin, vincristine, and prednisone
GCB: germinal center B-cell
DMSO: dimethylsulfoxide
SCID: severe combined immunodeficient
DEL: double expressor lymphoma
DDR: DNA damage repair
