# Supplementary material for: Preclinical efficacy and biological effects of the oral proteasome inhibitor ixazomib in diffuse large B-cell lymphoma
Source: Oncotarget. 2017 Aug 21;9(1):346–60. doi: 10.18632/oncotarget.20378 (PMC5787470; doi:10.18632/oncotarget.20378)
Supplement: Supplementary file 1 [file oncotarget-09-346-s001.pdf]

Preclinical efficacy and biological effects of the oral proteasome inhibitor ixazomib in diffuse large B-cell lymphoma

SUPPLEMENTARY MATERIALS

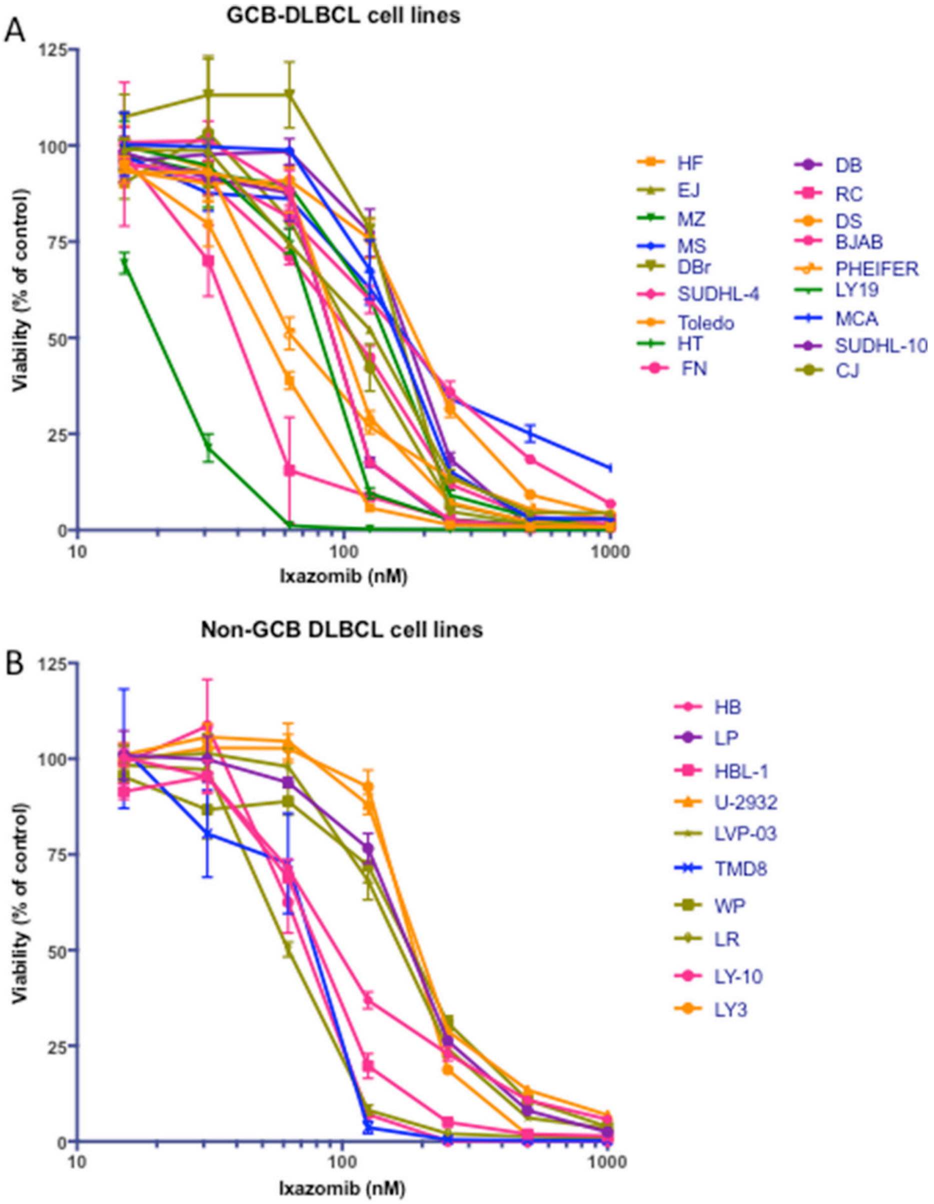

Supplementary Figure 1: *In vitro* efficacy of the proteasome inhibitor ixazomib in DLBCL. The effect of ixazomib at various drug concentrations for 72 h on viability of (**A**) 18 GCB- and (**B**) 10 Non-GCB-DLBCL cell lines was assessed.

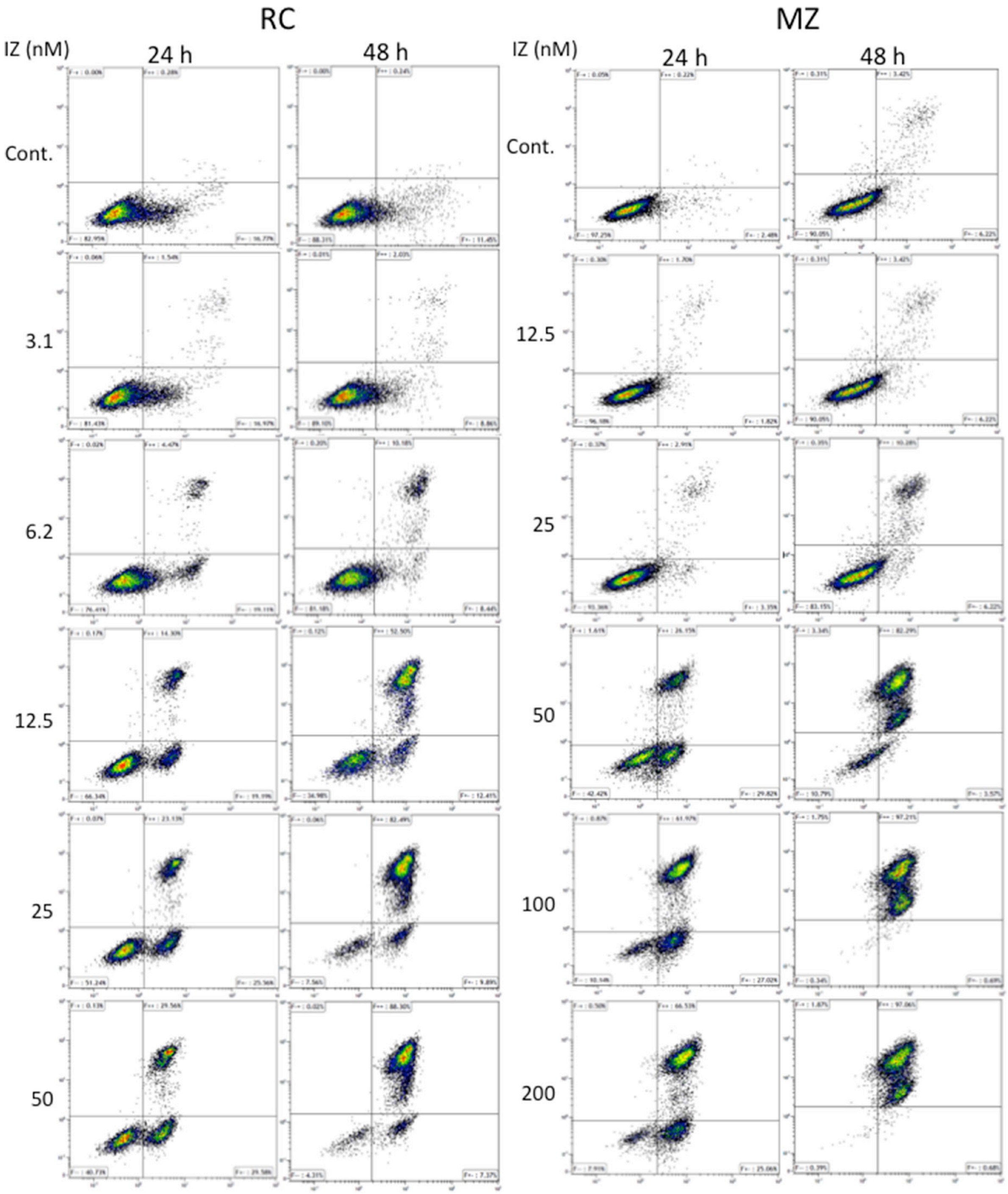

Supplementary Figure 2: Induction of apoptosis by ixazomib in DLBCL cells. Ixazomib-sensitive DLBCL cell lines RC (left panel) and MZ (right panel) were treated with ixazomib (IZ) at various doses for 24 or 48 h. Cells were then subjected to annexin V staining/ FACS analysis to identify those undergoing apoptosis. Histograms show increased apoptotic cells after ixazomib treatment.

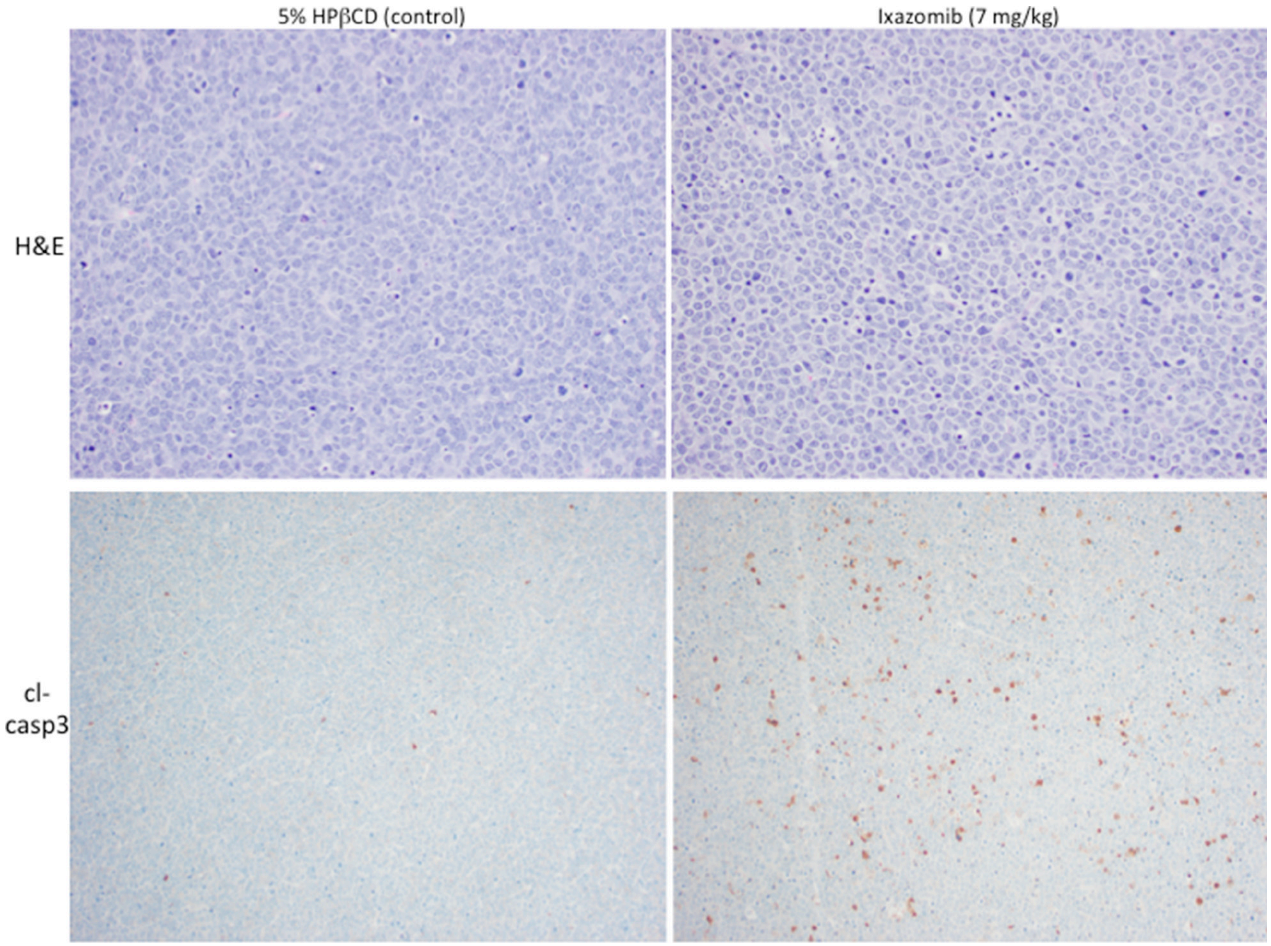

Supplementary Figure 3: Examination of caspase-3 activation in tumor tissues from control and ixazomib-treated mice. Top panels, H&E staining of tumor tissues from control and ixazomib-treated mice. Bottom panels, cleaved caspase-3 IHC staining of tumor tissues from control and ixazomib-treated mice. 10× images.

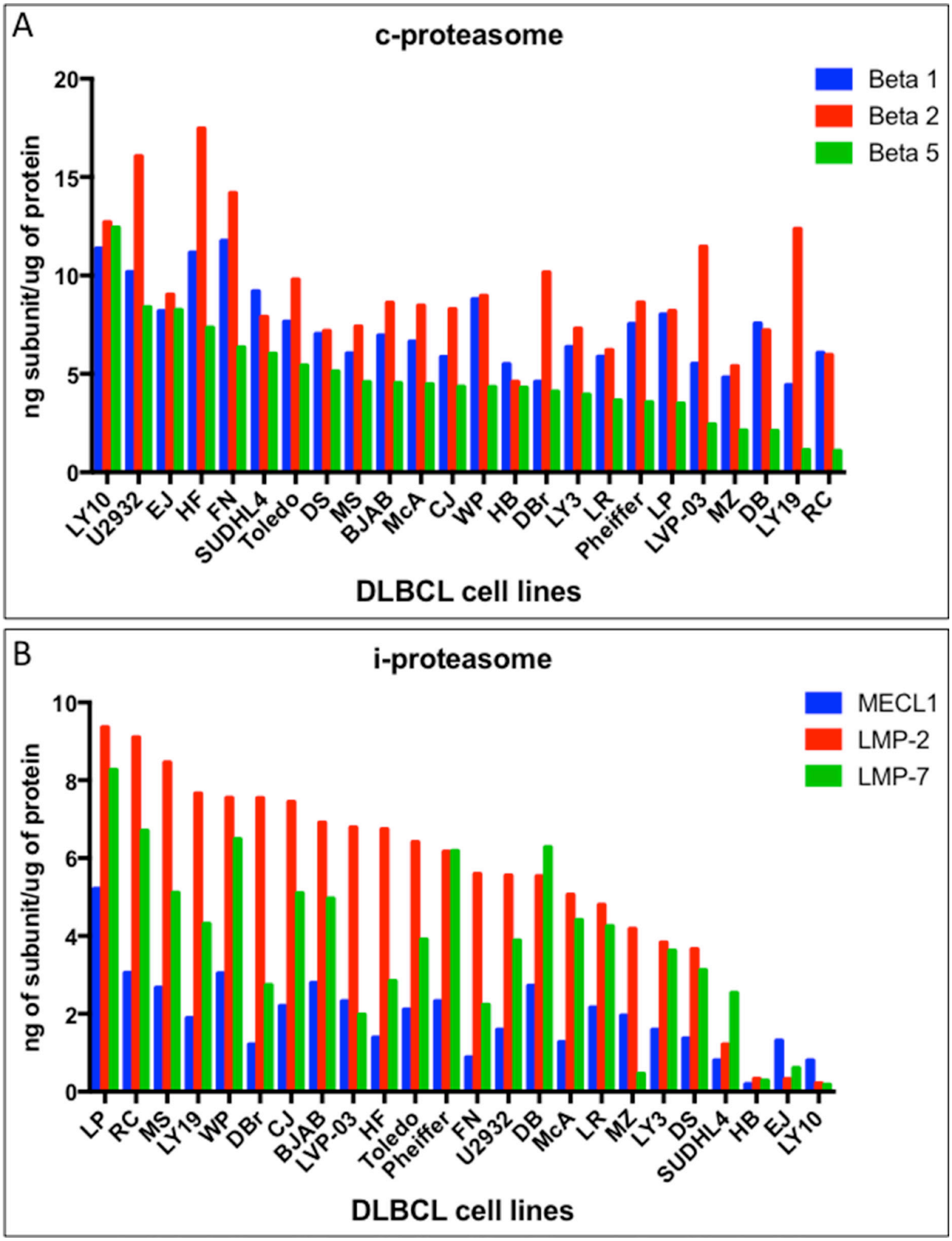

Supplementary Figure 4: ProCISE analysis for proteasomal activities in DLBCL cells. Proteasomal activities were assessed by ProCISE assay in 24 representative DLBCL cell lines. (**A**) Activity of constitutive proteasome (c-proteosome) subunits Beta1, Beta2, and Beta5. (**B**) Activity of immunoproteasome (i-proteosome) subunits MECL-1, LMP-2, and LMP-7.

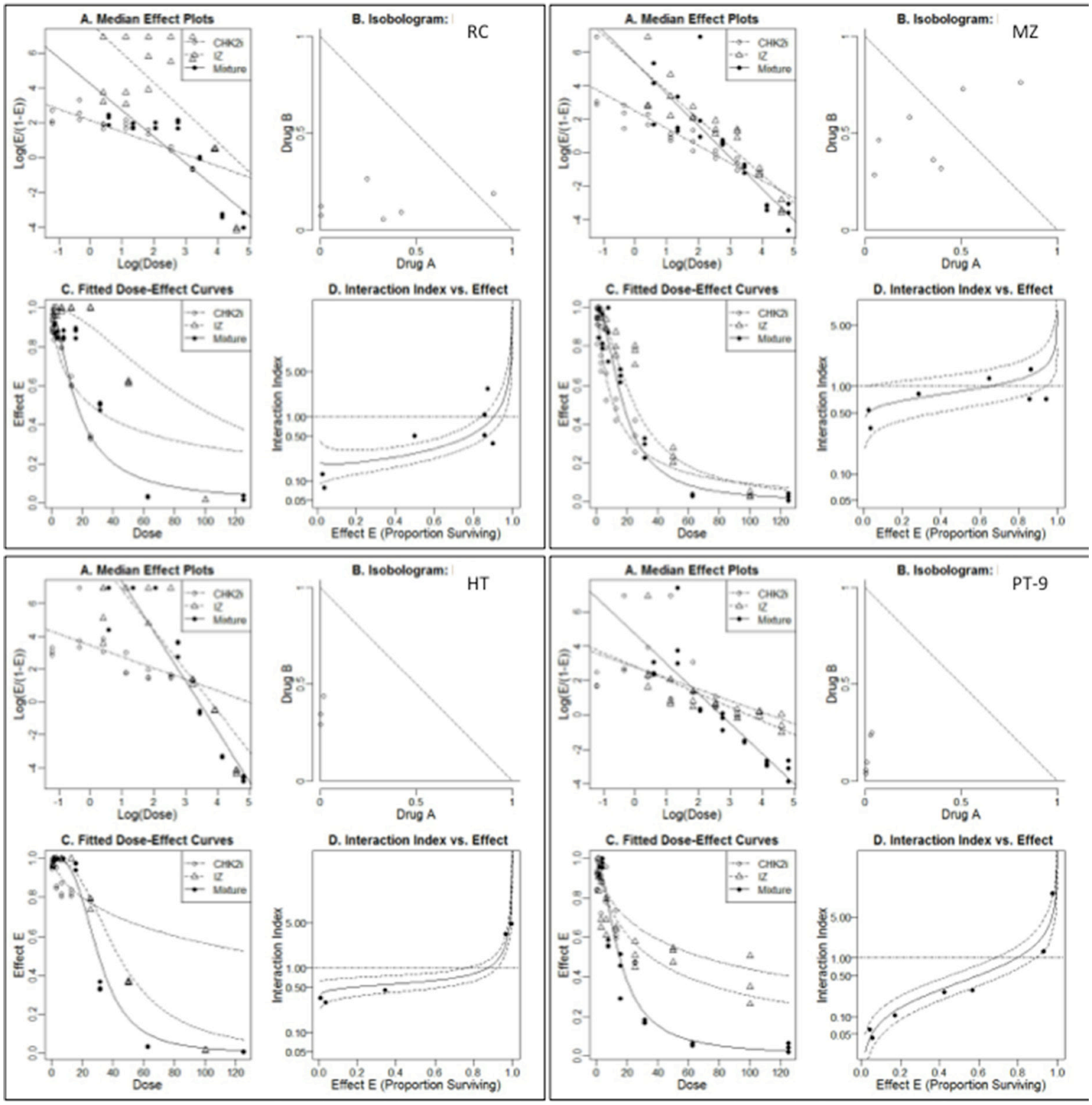

Supplementary Figure 5: Synergistic effects of CHK2 inhibitor and Ixazomib in 3 representative DLBCL cell lines (RC, MZ, and HT) and a primary DLBCL sample (PT-9). Median-effect plot (Panel **A**), isobologram (Panel **B**), plots of dose effect curves (Panel **C**) and combination indices versus effects (Panel **D**) for the combination doses at the fixed ray for CHK2i (Drug **A**) and ixazomib (Drug **B**). In Panel D, the solid line is the plot of the estimated combination indices versus effects (proportion cell surviving). Interaction index in panel D and combination index in panel B < 1 means synergy. The circles in Panel B from right to left, which gives the point estimates of the combinations in the isobologram, and the vertical bars in Panel D from left to right, which gives the 95% confidence intervals of the combination indices for observed combinations, correspond to the combination doses of (100, 25), (50, 12.5), (25, 6.25), (12.5, 3.1), (6.25, 1.5), (3.1, 0.75), (1.5, 0.3), respectively.
